# Perceptions of Plastic Surgery Training in the United Kingdom: A Mixed-Methods Trainee Survey

**DOI:** 10.7759/cureus.101294

**Published:** 2026-01-11

**Authors:** Areej-Noor Y Shah, Mohammad Anwar

**Affiliations:** 1 Medicine, University Hospitals Birmingham, Birmingham, GBR; 2 Burns, Mid Yorkshire Teaching NHS Trust, Wakefield, GBR

**Keywords:** plastic surgery, plastic surgery training, postgraduate training, surgical education, trainee satisfaction, uk

## Abstract

Background: Plastic surgery training in the United Kingdom (UK) is a highly competitive and evolving field. Recent reforms have aimed to standardise operative exposure, academic opportunities, and mentorship, yet the perspectives of current trainees remain underexplored.

Objective: This study assessed UK plastic surgery trainees’ perceptions of their training quality, satisfaction, and preparedness for independent practice.

Methods: A cross-sectional, anonymised online survey was distributed to UK plastic surgery trainees. The questionnaire included demographic items, views on training duration and fellowship structure, and satisfaction ratings across key domains of training using a five-point Likert scale. Free-text comments were thematically analysed to identify recurrent qualitative themes.

Results: Fourteen trainees from multiple UK deaneries responded. Half reported three to four years of training (n = 7, 50%), and four (29%) had less than one year of training. Most respondents (n = 9, 64%) believed six years of training was sufficient to achieve key competencies, while two (14%) disagreed and three (21%) were uncertain. Opinions on fellowship structure were divided: seven (50%) supported integration within the six-year programme (5+1), six (43%) preferred a separate post-CCT (certificate of completion of training) fellowship (6+1), and one (7%) was unsure. Mean overall satisfaction was 3.2/5 (median = 3). Highest domain scores were supervisor involvement (mean = 3.5/5, n = 14) and case variety/hands-on opportunities (mean = 3.5/5, n = 14); lowest were research opportunities (mean = 2.9/5, n = 14) and work-life balance (mean = 3.0/5, n = 14). Qualitative responses highlighted five themes: limited operative exposure, inconsistent mentorship, regional variation, restricted research time, and the lasting impact of COVID-19.

Conclusions: UK plastic surgery trainees report moderate satisfaction overall, with particular concern regarding research access and work-life balance. Qualitative feedback revealed recurring issues around mentorship and operative opportunities, consistent with national trainee trends.

## Introduction

Plastic surgery training in the United Kingdom (UK) is a six-year run-through programme governed by the Intercollegiate Surgical Curriculum Programme (ISCP) and aligned with the General Medical Council's (GMC) Excellence by Design standards for competency-based postgraduate education [1,2]. The framework aims to produce surgeons with balanced operative, academic, and professional skills through structured supervision and progressive responsibility.

Despite reforms, variability in training quality persists across UK deaneries. National evaluations and trainee-led surveys have identified disparities in supervision, operative exposure, and access to academic opportunities [3,4]. In the GMC’s 2025 National Training Survey of over 71,000 doctors and trainers, 61% of trainees considered themselves at moderate or high risk of burnout, and only 52% reported that they could always use their allocated time for training [5]. The COVID-19 pandemic further disrupted surgical education by reducing elective case numbers and delaying trainee progression [6].

Trainee-led surveys in plastic surgery have echoed these findings, reporting uneven teaching quality, regional inequality, and widespread intention to pursue additional fellowship training to achieve confidence in subspecialty practice [7]. Qualitative research also highlights challenges around mentorship, workload, and limited protected time for research, all of which affect trainee satisfaction and well-being [8].

While national reports like the National Training Survey (NTS) have assessed training outcomes across specialities, few contemporary studies have explored the lived experiences of plastic surgery trainees using both quantitative and qualitative approaches. Understanding current trainee perceptions is crucial for aligning future curriculum design with workforce expectations [9]. This study, therefore, aimed to evaluate UK plastic surgery trainees’ perceptions of their training, focusing on satisfaction across key domains, preparedness for independent practice, and areas for improvement within the national training framework.

## Materials and methods

Study design

A descriptive, cross-sectional mixed-methods study was conducted among UK plastic surgery trainees. An anonymised online survey was distributed via Google Forms (Google, Mountain View, CA) between July 2023 and January 2024. The survey link was circulated through trainee networks and social media platforms. Participation was voluntary and anonymous, with no identifiable data collected. According to the NHS Health Research Authority guidelines, ethical approval was not required for this service evaluation.

Survey instrument

The survey consisted of multiple-choice, Likert-scale (1-5) and open-ended free-text questions covering deanery and training duration; perceptions of the adequacy of six years of training and fellowship structure; satisfaction with supervisor involvement, operative exposure, mentorship and guidance, research opportunities, work-life balance, and overall training experience. The final item invited respondents to provide free-text comments or suggestions regarding improvements to training.

Data analysis

Quantitative Analysis

Numerical data were analysed using Python (pandas, matplotlib) (Python Software Foundation, Wilmington, Delaware). Descriptive statistics (mean, median, counts, percentages) were calculated. Likert-scale data were summarised as mean domain scores and visualised in bar charts.

Qualitative Analysis

Free-text comments were analysed using an inductive thematic analysis approach following Braun and Clarke’s six-step framework. Two authors independently reviewed responses, coded key ideas, and grouped them into overarching themes. Illustrative quotations were extracted to exemplify each theme.

## Results

Quantitative findings

Respondents

Fourteen trainees from multiple UK deaneries participated, representing a wide geographic distribution across the national training regions. The largest group were from Thames Valley and Wessex (n = 3, 21.4%), followed by Northern Ireland (n = 2, 14.3%). Single respondents were reported from East Midlands (n = 1, 7.1%), London (Pan-Thames) (n = 1, 7.1%), North East (n = 1, 7.1%), Scotland (n = 1, 7.1%), and Yorkshire (n = 1, 7.1%).

Training Duration

Four trainees (29%) had less than one year in the programme. One trainee (7%) had one to two years of experience. Half of the respondents were in the mid-stage of training, with seven trainees (50%) reporting three to four years of experience, and two (14%) were in the final five to six years of training.

Perception of Training Adequacy

Nine trainees (64%) felt six years was sufficient to achieve key competencies; two (14%) disagreed, and three (21%) were uncertain.

Fellowship Structure

Seven trainees (50%) supported integrating a fellowship within the six-year programme (5+1 model), six (43%) preferred a separate post-CCT (certificate of completion of training) fellowship (6+1), and one (7%) was unsure.

Satisfaction

**Figure 1:**
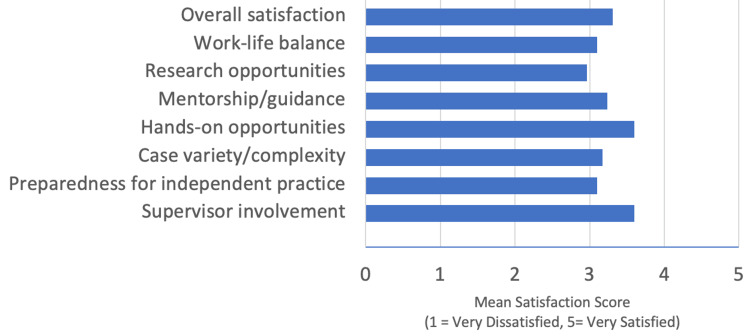
Mean satisfaction scores across training domains.

The mean overall satisfaction was 3.2/5 (median = 3, n = 14). Highest satisfaction scores were related to supervisor involvement (mean = 3.5/5, n = 14) and hands-on opportunities (mean = 3.5/5, n = 14). Lowest scores were research opportunities (mean = 2.9/5, n = 14) and work-life balance (mean = 3.0/5, n = 14) (Figure 1).

**Figure 2:**
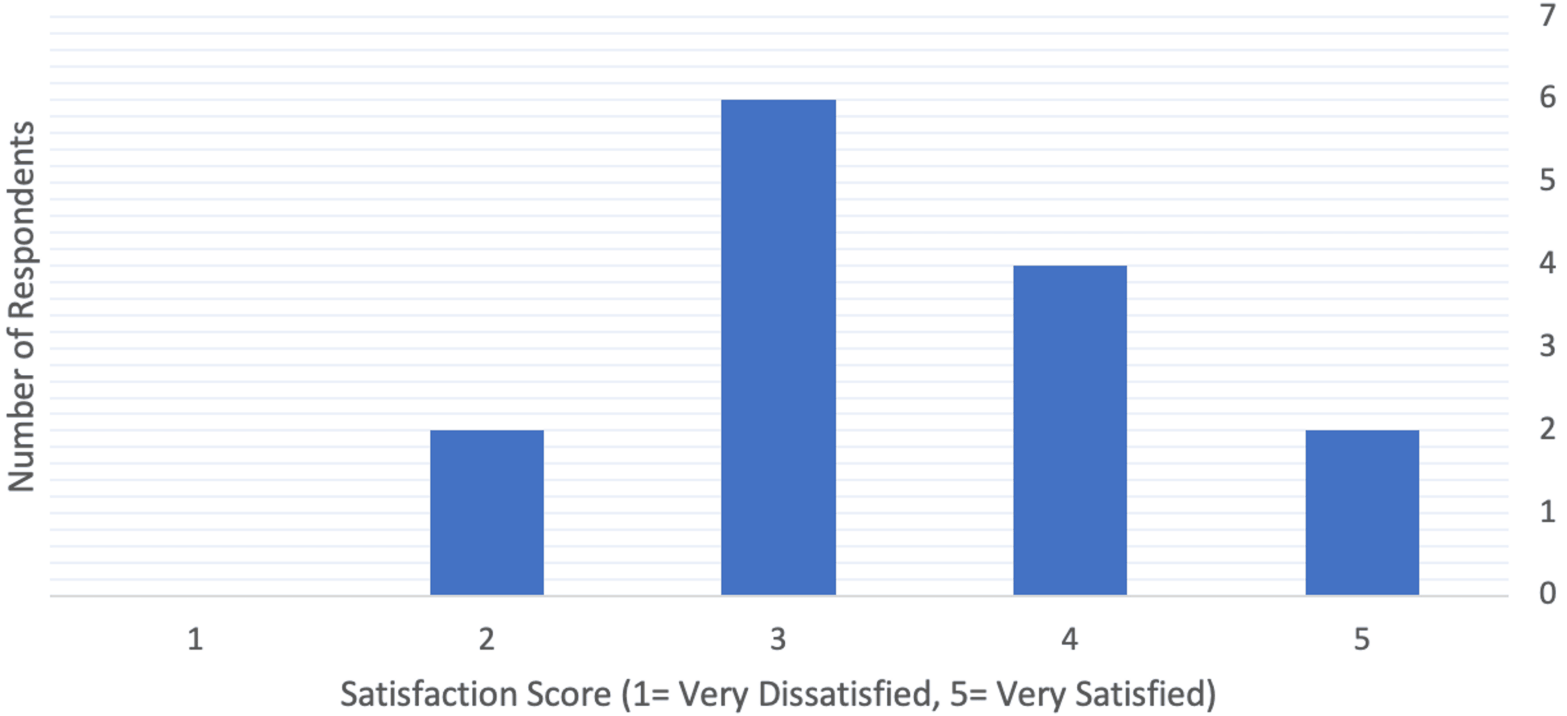
Distribution of overall satisfaction scores among UK plastic surgery trainees.

The distribution of overall satisfaction (Figure 2) showed clustering between scores 3 and 4, consistent with moderate satisfaction overall.

Qualitative findings

Free-text responses provided further insight into trainees’ experiences. Five key themes emerged. (1) Operative exposure: trainees described limited access to complex reconstructive procedures and persistent COVID-related reductions in case numbers. (2) Supervision and mentorship: comments reflected inconsistent mentorship and a need for structured consultant engagement. (3) Regional variation: trainees noted differences in operative exposure and teaching between deaneries. (4) Research opportunities: lack of protected research time was a consistent concern. (5) Workload and well-being: high service demands and poor work-life balance were recurrent. Representative quotations are shown in Table 1.

**Table 1:** The five major qualitative themes emerging from trainee feedback, with corresponding descriptions and illustrative quotations.

Theme	Description	Illustrative quote
Operative exposure	Concern over limited access to complex reconstructive cases, exacerbated by service pressures and COVID-19 recovery.	“Far too much service provision. Not nearly enough educational activity (cadaver labs, tutorials). Not enough supervised operating.”
Supervision & mentorship	Strong desire for more structured, hands-on guidance and formalised mentorship.	“Trainers should consider their role as one of a mentor[…] Without adequate supervision and support, overall quality of training is declining.”
Regional variation	Recognition that the quality of training differs widely across deaneries, with some regions offering broader exposure.	“[Redacted] is a brilliant deanery with almost all subspecialties, high-quality surgeons & trainers.”
Research opportunities	Limited protected time for academic activity due to service demands.	“No time within the training day to complete any research or portfolio work.”
COVID-19 impact	The pandemic’s long-term effect on operative exposure and confidence.	“Lack of operating theatre access after COVID has severely restricted trainee access to key cases.”

Qualitative data reinforced quantitative findings, highlighting variability in mentorship, the burden of service provision, and ongoing barriers to academic participation.

## Discussion

This mixed-methods survey provides an updated snapshot of UK plastic surgery trainees’ perceptions of their training quality and readiness for independent practice. Overall satisfaction among respondents was moderate (mean = 3.2/5), with the highest scores for supervision and operative exposure but lower ratings for research opportunities and work-life balance. These findings reflect persistent systemic challenges previously described in national reports and align with broader trends across UK surgical training [3-5,7-9].

Training adequacy and structure

Most respondents (n = 9, 64%) agreed that the six-year run-through programme provides sufficient time to achieve core competencies, consistent with the competency-based framework outlined in the ISCP Plastic Surgery Curriculum (2025) and the GMC’s Excellence by Design standards [1,2]. However, divided opinions about fellowship integration mirror findings from previous national surveys, where many trainees reported plans for post-CCT fellowships to consolidate subspecialty confidence [7,9].

The Royal College of Surgeons (RCS) of England’s Improving Surgical Training evaluation (2015) noted that while outcome-based curricula streamline progression, consistent access to complex index cases remains crucial for full speciality breadth [3]. The perception that six years achieves competency but not mastery reinforces the need for targeted exposure to subspecialty procedures and for fellowship pathways to remain accessible.

Supervision, mentorship, and educational support

High satisfaction with supervision (mean = 3.5/5) indicates strong consultant engagement; however, qualitative feedback highlights variation in the quality and structure of mentorship. Cooper et al. (2022) reported similar inconsistencies across deaneries and recommended formal mentorship schemes and protected time for trainers [8]. The Plastic Surgery Trainees Association (PLASTA) National Training Survey (2021) also found that over 80% of plastic surgery trainees lacked access to structured mentoring [10]. Establishing protected educational time and formal mentor-mentee pairings would help ensure consistency and equity between deaneries.

Regional variability remains a central theme. The GMC National Training Survey 2025 reported that only 52% of all UK trainees could always use their allocated training time, and 61% were at moderate or high risk of burnout, illustrating systemic pressures that may limit educational quality across regions [5]. Transparent benchmarking of deanery-level training outcomes, similar to mechanisms advocated in the RCS (2015) report, could mitigate inequities by identifying under-performing units early [3].

Operative exposure and the impact of COVID-19

Operative experience remains a cornerstone of surgical training. Respondents described lingering reductions in case exposure following COVID-19. This aligns with Ibrahim et al. (2021), who documented major declines in operative opportunities and perceived competency during pandemic restrictions [6]. While elective services have largely resumed, exposure to complex reconstructive cases is uneven, reinforcing the value of simulation-based training and equitable case distribution. The ISCP (2025) curriculum’s inclusion of entrustable professional activities provides a framework to monitor competence irrespective of regional variation [2].

Research engagement and academic development

Research opportunity was rated lowest (mean = 2.9/5). Clement et al. (2023) demonstrated that structured trainee research collaboratives enhance participation and productivity through shared infrastructure, mentorship, and administrative support [11]. Extending such frameworks to plastic surgery could address the consistent barriers identified by Fell et al. (2020) and by trainees in this study, i.e., limited protected time and competing clinical workload [7]. National bodies such as PLASTA are well-positioned to coordinate collaborative, multicentre trainee-led research consistent with curricular objectives [10].

Work-life balance and well-being

Trainees consistently highlighted the impact of workload on well-being. The GMC NTS 2025 found that burnout risk remains high, with a significant proportion of trainees reporting reduced morale [5]. Similar trends were confirmed in the systematic review by Balendran et al. (2021), which identified high burnout prevalence across UK surgical specialities and linked it to excessive service pressures and inadequate work-life balance [12]. Addressing this requires structural change: enforcing protected training time, enabling less-than-full-time (LTFT) flexibility, and embedding well-being initiatives. The LTFT qualitative study in plastic surgery supports this, noting that flexible training improves retention and well-being without reducing training quality [13].

Regional variation

Marked inter-deanery differences in exposure, mentorship, and academic support align with previous reports [4,5]. Addressing this inequity requires national benchmarking of training opportunities and transparent reporting of key performance indicators, including operative log numbers, teaching hours, and trainee-to-trainer ratios.

The RCS (2015) has called for regional training boards to use NTS data more proactively to identify outlier units and provide targeted support [3]. Localised mixed-methods audits, such as the present study, can complement these national mechanisms by highlighting specific contextual issues that broad surveys may overlook.

Broader implications

These findings have practical implications. First, they reinforce that trainee satisfaction depends on both the structure of curricula and the lived reality of training environments. Policies that safeguard training time, standardise mentorship, and support research engagement are vital. Second, integrating local trainee feedback, such as that collected in this survey, into national review cycles (e.g., GMC NTS and PLASTA surveys) can provide real-time monitoring of curricular impact [5,10,14,15].

Finally, the emerging PLASTA 2025 National Training Survey initiative underscores the increasing role of trainee-led data collection in shaping policy [10,14]. Combining these datasets with national oversight (GMC, ISCP, RCS) would allow for a longitudinal evaluation of how recent reforms influence competency attainment, well-being, and career intentions in plastic surgery.

Limitations

The strengths of this study include its mixed-methods design and qualitative depth. Limitations include its small, voluntary sample (n = 14) and potential response bias. Nevertheless, the alignment between these findings and larger national reports supports the study’s validity as an exploratory snapshot of current trainee sentiment.

Future directions

Key recommendations arising from this analysis include the development of a national mentorship framework that ensures protected time for trainers and structured support for trainees [8,12]. Expansion of trainee-led research collaboratives, coupled with the provision of protected academic time, would help promote equitable access to research opportunities [7,10,12]. Transparent benchmarking and reporting of operative exposure and mentorship quality between deaneries are essential to enhance accountability and drive consistency in training standards [3,5]. Embedding LTFT training options and well-being initiatives within postgraduate curricula would further strengthen trainee welfare and flexibility [11,13]. Finally, integrating locally driven trainee surveys into regular national quality-assurance processes would enable continuous feedback and data-informed improvement of training programmes [5,12,14,15].

## Conclusions

This study highlights that while UK plastic surgery trainees generally feel supported in their supervision and operative exposure, significant challenges remain in achieving consistent mentorship, equitable access to research opportunities, and a sustainable work-life balance. Trainees continue to perceive variability in training quality across regions and often view additional post-CCT fellowships as necessary to consolidate their skills and confidence.

Improving these areas will require more structured mentorship, protected time for research and training, and greater standardisation of educational opportunities between deaneries. Prioritising trainee well-being, ensuring equitable access to operative experience, and maintaining responsive feedback mechanisms will be vital to creating a modern, balanced, and resilient surgical training system that supports both excellence in patient care and the professional development of future plastic surgeons. Given the exploratory nature of this study and its small, voluntary sample, further research with larger and more representative cohorts will be important to validate these findings and inform national training policy.
